# Bovine Milk and Yogurt Affect Oral Microorganisms and Biofilms In-Vitro

**DOI:** 10.3290/j.ohpd.b3920023

**Published:** 2023-02-24

**Authors:** Christian Tennert, Giada Sarra, Alexandra Stähli, Anton Sculean, Sigrun Eick

**Affiliations:** a Assistant Professor, Department of Preventive, Restorative and Pediatric Dentistry, University of Bern, School of Dental Medicine, Bern, Switzerland. Idea, hypothesis, financial support, wrote the manuscript, contributed substantially to discussion.; b Dentist, Department of Preventive, Restorative and Pediatric Dentistry, University of Bern, School of Dental Medicine, Bern, Switzerland. Department of Periodontology, University of Bern, School of Dental Medicine, Bern, Switzerland. Performed the experiments, contributed substantially to discussion.; c Periodontist, Department of Periodontology, University of Bern, School of Dental Medicine, Bern, Switzerland. Contributed substantially to discussion.; d Professor and Chair, Department of Periodontology, University of Bern, School of Dental Medicine, Bern, Switzerland. Proofread the manuscript.; e Professor, Department of Periodontology, University of Bern, School of Dental Medicine, Bern, Switzerland. Idea, hypothesis, experimental design, wrote the manuscript, consulted on and performed statistical evaluation.

**Keywords:** Candida, caries, milk products, oral bacteria, periodontitis

## Abstract

**Purpose:**

To evaluate the effect of bovine milk and yogurt on selected oral microorganisms and different oral biofilms.

**Materials and Methods:**

Milk was prepared from 0.5% fat (low-fat) and 16% fat (high fat) milk powder. For yogurt preparation, the strains *Lactobacillus delbrueckii ssp. bulgarcius* and* Streptococcus thermophilus* were added to the milk. Minimal inhibitory concentrations (MIC) and minimal microbiocidal concentrations (MMC) of the test compounds were measured against various microorganisms by the microbroth dilution technique. Cariogenic periodontal biofilms and one containing Candida were created on plastic surfaces coated with test substances. Further, preformed biofilms were exposed to the test substances at a concentration of 100% for 10 min and thereafter 10% for 50 min. Both colony forming units (cfu) and metabolic activity were quantified in the biofilms.

**Results:**

Neither high-fat milk, low-fat milk nor casein inhibited the growth of any species. Yogurt and *L. delbrueckii ssp. bulgaricus* at low MIC and MMC suppressed the growth of *Porphyromonas gingivalis* and other bacteria associated with periodontal disease. High-fat yogurt decreased cfu in the forming periodontal biofilm by 90%. Both low- and high-fat yogurts reduced metabolic activity in newly forming and preformed periodontal and Candida biofilms, but not in the cariogenic biofilm.

**Conclusions:**

Yogurt and *L. delbru eckii ssp. bulgaricus,* but not milk, were bactericidal against periodontopathogenic bacteria. Yoghurt reduced the metabolic activity of a Candida biofilm and a periodontal biofilm. Yogurt and *L. delbrueckii ssp. bulgaricus* may have potential in prevention and therapy of periodontal diseases and Candida infections.

Milk is the first food in mammals and provides energy and nutrients that are essential for growth and development. After weaning, the consumption of milk is discontinued except in humans, who continue to consume milk and dairy products during adulthood.^30^

Nutritional associations recommend milk and dairy products as an important source of protein and calcium in our diet.^3^ According to the Food and Agriculture Organization of the United Nations, FAO, about 6 billion people worldwide consume milk and dairy products. Bovine milk and its products are the predominant dairy products consumed today, accounting for about 85% of the total milk products consumed. Milk is rich in many different important nutrients such as lipids, carbohydrates, minerals and vitamins. On average, bovine milk consists of 87% water, 4–5% lactose, 3% protein, 3%–4% fat, 0.8% minerals, and 0.1% vitamins.^30^ Milk is an important source of proteins which can be divided into different groups, with casein accounting for 78% of protein content in bovine milk.^14^ Besides their nutritional value, the bioactive properties of the different milk proteins (α-lactalbumin, β-lactoglobulin, α-casein, β-casein, and κ-casein) play an important role. For instance, the antimicrobial activity of some of these peptides can prevent the growth of bacteria.^31^

Yogurt is a dairy product in which pasteurised milk is fermented with *Streptococcus salivarius ssp. thermophilus* and *Lactobacillus delbrueckii ssp. bulgaricus,* thereafter reaching a pH of about pH 4.5.^2^

A systematic review evaluated the influence of various beverages, e.g. milk, coffee, alcoholic beverages, tea and sugary drinks, on oral health in aged populations.^43^ Intake of alcoholic and sugary beverages were associated with tooth loss, whereas milk and coffee negatively influenced the development of periodontal diseases.^43^

An epidemiologic study in nearly 7000 children and adolescents showed that those who consumed a high quantity of yogurt and a certain amount of cheese had a lower risk of caries.^40^

Intake of yogurt was negatively correlated with the prevalence of periodontitis in a Korean population.^40^ A follow-up study over five years in Japan found that the consumption of yogurt was associated with a reduced risk of tooth loss due to periodontal disease.^18^ The authors of these two studies discuss the beneficial effects of yogurt with modified microbiota in the oral biofilms.^17,18^

Oral bacteria and biofilms are associated with the initiation and progression of caries and periodontitis. Caries is the result of an ongoing ecological shift of the dental biofilm from homeostasis to dysbiosis. The main driver of this dysbiotic shift is frequent consumption of sugars. The lower pH generated from the metabolisation of sugars through cariogenic bacteria impels the selection of these acid-producing and acid-loving species at the expense of the beneficial oral bacteria that prefer an approximately neutral pH (homeostasis).^34^

The aetiology of periodontitis is thought of as an interaction of the host response with a dysbiotic biofilm.^9^ In development of a dysbiotic biofilm and modifying host response, *Porphyromonas gingivalis* plays a key role.^13^ *Candida albicans*, normally a commensal in oral microbiota, can lead to infection when the immune response is compromised. Under such conditions, its numbers increase, virulence factors are synthesised in higher quantities, and it forms biofilms.^29^

Although the beneficial effect of milk and yogurt has been reported, in-vitro data on activity against oral microorganisms and biofilms are scarce. Milk casein inhibited adhesion of *S. mutans* to saliva-coated hydroxyl apatite.^5^ Yogurt decreased counts of *S. mutans* by about 90%, but was less active against non-mutans streptococci.^32^ A probiotic yogurt (containing *L. bulgaricus, S. thermophilus*, and supplemented with* L. acidophilus* and *Bifidobacterium*) inhibited all investigated periodontal pathogens.^42^

The aim of this study was to evaluate the effect of bovine milk and yogurt on selected oral microorganisms and different biofilms – cariogenic biofilm, periodontal biofilm and Candida biofilm. The hypothesis was that bovine milk and related dairy products might inhibit microorganisms and biofilms associated with caries, periodontal disease and Candida infection.

## Materials and Methods

### Milk and Yogurt Preparation and Related Test Compounds

The milk and the yogurt were prepared from two milk powders (Nestle Stalden and Nestle Stalden Viva, Nestle; Vevey, Switzerland) using sterile tap water according to the manufacturer’s recommendation. This resulted in two types of milk, one with 0.5% fat (low-fat) and the other with 16% fat (high fat).

For yogurt preparation, the strains *Lactobacillus delbrueckii ssp. bulgarius* and *Streptococcus thermophilus* (My.Yo, Metafood; Frankfurt, Germany) were cultured on agar plates and suspended in 0.9% w/v NaCl to McFarland 0.5. 0.5 ml of the suspension was added to 50 ml of each milk type prepared as mentioned above and preheated to 37°C. The yogurt was made in a yogurt maker (Gaia Joghurtmaker, Klarstein, Chal-Tec; Zürich, Switzerland) according to the manufacturer’s protocol.

In the minimal inhibitory concentrations (MIC) and minimal microbiocidal concentrations (MMC), the strains *L. delbrueckii ssp. bulgaricus* and *S. thermophilus* adjusted to McFarland 0.5 in 0.9% w/v NaCl were used as test compounds.

As the major protein in bovine milk, casein complemented the test substances in the MIC/MMC tests. The starting concentration was 26 mg/ml casein from bovine milk (Merck; Darmstadt, Germany).

### Microorganisms

The test strains used in the experiments are shown in Table 1. Most strains were reference strains, but four clinical isolates stored in the strain collection of the University of Bern or Basel were also included. The MICs and MMCs of the test compounds were measured against all strains. Three biofilms were created: a cariogenic, a periodontal and a Candida-containing biofilm. For the respective biofilms, certain strains were selected (Table 1).

**Table 1:** Strains used in the assays

Species	Strain number	Used in biofilm assays
*Streptococcus gordonii*	ATCC 10558	All
*Streptococcus sanguinis*	ATCC 10556	
*Streptococcus mutans*	ATCC 25175	Cariogenic
*S. mutans*	ZIB 1583^*^	
*S. mutans*	ZIB 6551^*^	
*Streptococcus sobrinus*	ATCC 33478	Cariogenic
*Actinomyces naeslundii*	ATCC 12104	All
*Lactobacillus acidophilus*	ATCC 11975	Cariogenic
*Fusobacterium nucleatum*	ATCC 25586	Periodontal
*Parvimonas micra*	ATCC 33270	Periodontal
*Porphyromonas gingivalis*	ATCC 33277	Periodontal
*P. gingivalis*	Be-TR 4/5^*^	
*P. gingivalis*	Be-TR 602/9^*^	
*Tannerella forsythia*	ATCC 43037	Periodontal
*Candida albicans*	ATCC 76615	Candida

^*^Clinical isolates, all other strains are laboratory strains.

Before performing the experiments, the strains were precultivated on tryptic soy agar plates (supplemented with 5% sheep blood [Oxoid; Basingstoke, UK]) in the respective atmosphere (streptococci, *A. naeslundii* in 10% CO_2_; Candida aerobically, others anaerobically) at 37°C.

### Minimal Inhibitory and Minimal Microbiocidal Concentrations

The antimicrobial activity of the test substances against the various microorganisms was tested by the microbroth dilution technique in 96-well microtiter plates.

First, from the test products, a two-fold dilution series was prepared with 0.9% w/v NaCl. Then, 50 µl of each of the test substances suspensions were pipetted per well. NaCl served as a control. The microorganisms were adjusted to a McFarland of 0.5 in NaCl, then mixed 1:10 with culture medium as follows: streptococci, cation-adjusted Mueller-Hinton broth (Oxoid); Candida, RPMI 1640 medium (ThermoFisher Scientific; Waltham, MA, USA) with 2% of glucose; others, Wilkins-Chalgren broth (Oxoid) supplemented with 10 µg/ml β-NAD. From the suspension, 50 µl were then added to each well. The plates were incubated in the respective atmosphere. Subsequently, a subcultivation was done, MIC was defined as the lowest concentration clearly inhibiting growth and MMC as the lowest concentration with no more colonies visible. In the analyses, colonies of the yogurt strains were not included.

The experiments were conducted in independent replicates.

### Biofilms

In the experiments on biofilm formation, 96-well plates were coated with 12.5 µl milk or yogurt mixed 1:1 with 0.9% w/v NaCl per well and incubated for 30 min. Then, 12.5 µl of 1.5% bovine serum albumin (BSA)/0.67% mucin in PBS were added and the plates were incubated for another 30 min. The microorganisms of the respective biofilms were suspended in 0.9% NaCl and adjusted to McFarland 0.5. Thereafter, one part *S. gordonii* was mixed with each four parts of the remaining microorganisms. Next, culture medium (cariogenic biofilm: brain-heart infusion broth [BHI, Oxoid] with 0.5% glucose; periodontal biofilm: Wilkins-Chalgren broth with 10 µg/ml β-NAD; Candida biofilm: BHI mixed 1:1 with RPMI 1640) was added to the microbial suspension at a ratio of 9:1. 225 µl of the corresponding mixture (cariogenic, periodontal and candida biofilm) were pipetted per well. The microtiter plates were incubated for 6 h (cariogenic biofilm) and 24 h (periodontal and Candida biofilms). The cariogenic and Candida microbiota were cultured with 10% CO_2_, whereas the periodontal biofilm was anaerobically cultured. Then, after a short, careful washing, the biofilms were scraped from the surface and suspended in 0.9% w/v NaCl until an almost homogenous solution was reached. Aliquots were plated on agar plates, and finally the colony forming units (cfu) were counted. In addition, metabolic activity was quantified using Alamar blue.^33^

For experiments on established biofilms, a similar methodology was used as described in the following. The microtiter-plates were coated only with the BSA/mucin solution. Microbial suspension was added to culture media and 250 µl were pipetted per well. The incubation times were 48 h for the cariogenic and 3.5 days for the periodontal and Candida biofilms. In the case of the caries biofilm, 150 µl of medium was removed after 24 h and fresh medium was added. For the periodontal biofilm, *P. gingivalis* and *T. forsythia* were added again after 3.5 days. After the incubation period, the medium was carefully removed and 25 µl of each test substance was added. After 10 min, 225 µl of freshly prepared medium was added and the plates were incubated for another 50 min in the respective atmosphere. Thereafter, the remaining biofilms were analysed as described above.

Each experiment was conducted in two series including each independent quadruplicate per group and time-point. The respective mean values were compared via ANOVA with the post-hoc Bonferroni adjustment using SPSS 28.0 (IBM; Armonk, NY, USA).

## Results

### Minimum Inhibitory Concentration and Mininum Microbiocidal Concentration

The MICs and MMCs of each test substance were determined against all included microbial strains. Neither high-fat milk, low-fat milk (0.5%) nor casein inhibited the growth of any species. *Candida albicans* and the reference strain of *S. mutans* (ATCC 25175) were not affected by the test substances. Yogurt at relatively high concentrations inhibited the growth of the other included streptococci, i.e. *A. naeslundii* ATCC 12104 and *L. acidophilus* ATCC 11975. Yogurt at concentrations of about 1% inhibited the growth of the bacteria associated with periodontal diseases (*P. gingivalis, T. forsythia, F. nucleatum, P. micra*). *Lactobacillus delbrueckii ssp. bulgaricus* at low concentrations suppressed the growth of *P. gingivalis, T. forsythia* ATCC 43037, and *F. nucleatum* ATCC 25586. *S. thermophilus* inhibited only the reference strain of *P. gingivalis*. It became apparent that either a bactericidal activity already existed at low concentrations or there was no effect. Except for the low-fat and high-fat yogurt against the clinical isolates of *S. mutans,* there was no difference between the MIC and MMC values. The results are presented in Table 2.

**Table 2:** Minimal inhibitory and microbiocidal concentrations of milk, yogurt and related test compounds against oral microorganisms

Strain	Milk 16%	Milk 0.5%	Yogurt 0.5%	Yogurt 16%	Casein	*S. thermophilus* ^*^	*L. delbrueckii ssp bulgaricus* ^*^
*S. gordonii* ATCC 10588	>50%	>50%	50%	12.5%	>50%	>50%	>50%
*S. sanguinis* ATCC 10556	>50%	>50%	12.5%	25%	>50%	>50%	>50%
*S. mutans* ATCC 25175	>50%	>50%	>50%	>50%	>50%	>50%	>50%
*S. mutans* ZIB 1583	>50%	>50%	50% [>50%]	50% [>50%]	>50%	>50%	>50%
*S. mutans* ZIB 6551	>50%	>50%	50% [>50%]	12.5% [50%]	>50%	>50%	>50%
*S. sobrinus* ATCC 33478	>50%	>50%	>50%	25%	>50%	>50%	>50%
*A. naeslundii* ATCC 12104	>50%	>50%	6.25%	6.25%	>50%	>50%	>50%
*L. acidophilus* ATCC 11975	>50%	>50%	>50%	12.5%	>50%	>50%	>50%
*F. nucleatum* ATCC 25586	>50%	>50%	≤0.78%	0.78%	>50%	>50%	≤0.78%
*P. micra* ATCC 33270	>50%	>50%	≤0.78%	1.56%	>50%	>50%	>50%
*P. gingivalis* ATCC 33277	>50%	>50%	≤0.78%	≤0.78%	>50%	≤0.78%	≤0.78%
*P. gingivalis* Be-TR 4/5	>50%	>50%	≤0.78%	≤0.78%	>50%	>50%	≤0.78%
*P. gingivalis* Be-TR 602/9	>50%	>50%	≤0.78%	≤0.78%	>50%	>50%	≤0.78%
*T. forsythia* ATCC 43037	50%	50%	≤0.78%	≤0.78%	50%	>50%	≤0.78%
*C. albicans* ATCC 76615	>50%	>50%	>50%	>50%	>50%	>50%	>50%

^*^Tested as McFarland 0.5.

### Cariogenic Biofilm

**Fig 1:**
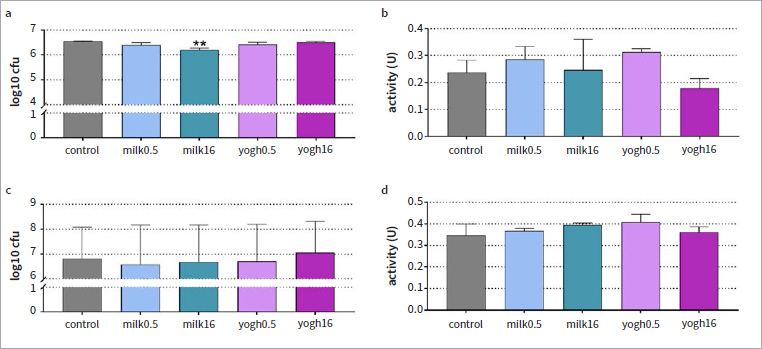
Colony counts (log10) (a, c) and metabolic activity (b, d) in the formed cariogenic biofilm for 6 h (a, b) and in a pre-formed biofilm (c, d) after exposure to milk (0.5% and 16%) and yogurt (0.5% and 16%). Biofilm formation: coating of the surface with test substances before biofilm formation, final concentration in the assay 2.5%; preformed biofilm: exposure of the 48-h-old biofilm to the test substances at a concentration of 100% for 10 min and thereafter 10% for 50 min. **p < 0.01 vs control.

The formation of the cariogenic biofilm was slightly influenced by high-fat milk. Statistically significant differences existed between the cfu counts, with a reduction by 0.36 log10 (equivalent to 22.9%) (p < 0.001, Fig 1a). There was no statistically significant difference in metabolic activity between the groups (Fig 1b).

When the biofilm had been grown for 48 h, application of milk and yogurt did not change the cfu counts (Fig 1a) or the metabolic activity (Fig 1b).

### Periodontal Biofilm

**Fig 2:**
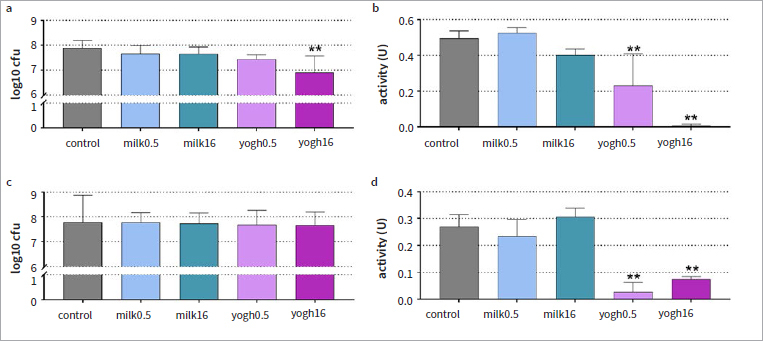
Colony counts (log10) (a, c) and metabolic activity (b, d) in the pre-formed periodontal biofilm for 24 h (a, b) and in a pre-formed biofilm (c, d) after exposure to milk (0.5% and 16%) and yogurt (0.5% and 16%). Biofilm formation: coating of the surface with test substances before biofilm formation, final concentration in the assay 2.5%; preformed biofilm: exposure of the 3.5-day-old biofilm to the test substances at a concentration of 100% for 10 min and thereafter 10% for 50 min. **p < 0.01 vs control.

When the surface was precoated with milk or yogurt, statistically significant differences were found for cfu und metabolic activity between the groups after 24 h. When comparing each test-substance group with the control group, it was found that cfus decreased by 0.97 log10 (equivalent to 93.3%) in the presence of high-fat yogurt vs control (<0.001, Fig 2a). Compared to the control, metabolic activity was also inhibited in the presence of low-fat and high-fat yogurt (p = 0.005 and p < 0.001, respectively; Fig 2b).

The cfu counts of the 3.5-day-old biofilm were not influenced by milk or yogurt (Fig 2c). Both low-fat and high-fat yogurt statistically significantly reduced the metabolic activity of the pre-formed periodontal biofilm (each p < 0.001 vs control, Fig 2d).

### Candida Biofilm

**Fig 3:**
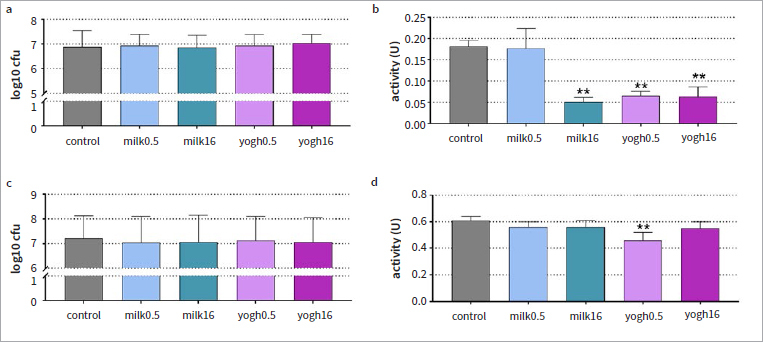
Colony counts (log10) (a, c) and metabolic activity (b, d) in the formed Candida biofilm for 24 h (a, b) and in a pre-formed biofilm (c, d) after exposure to milk (0.5% and 16%) and yogurt (0.5% and 16%). Biofilm formation: coating of the surface with test substances before biofilm formation, final concentration in the assay 2.5%; pre-formed biofilm: exposure of the 3.5-day-old biofilm to the test substances at a concentration of 100% for 10 min thereafter 10% for 50 min. **p < 0.01 vs control.

For Candida biofilm formation, no statistically significant differences were found in the comparison of the cfu mean values between the test groups and the control (Fig 3a). However, a statistically significant difference was found in the metabolic activity of the biofilms with high-fat milk, low-fat yogurt and high-fat yogurt compared to the control group (each p < 0.001). No statistically significant change in metabolic activity was observed with low-fat milk (Fig 3b).

There was also no change in the cfu counts after applying milk or yogurt to the 3.5-day-old biofilm (Fig 3c). The low-fat yogurt reduced the metabolic activity of the biofilm (p = 0.002, Fig 3d).

## Discussion

The present study has shown that yogurt and *L. delbrueckii ssp. bulgaricus* but not milk were bactericidal against periodontopathogenic bacteria and that yoghurt inhibited the metabolic activity of Candida and periodontal biofilms.

Yogurt is a fermented form of milk. Fermentation of lactose with *Lactobacillus bulgaricus* results in the production of lactic acid and a drop in pH. The fermentation also leads to a major reduction of the lactose content, leaving some galactose. However, the other components remain unchanged.^41^

Milk, yogurt and some of their naturally occuring ingredients against cariogenic had very limited activity against bacteria and biofilms. A very minor effect of high-fat milk on bacterial counts in biofilm was found. Milk contains substances which act preventively against bacterial adhesion. Milk casein was found to inhibit bacterial adherence to the pellicle and to decrease glycosyltransferase activity, thus reducing the formation of glucan.^23,38^ This might limit bacterial growth and biofilm formation. Guggenheim et al^12^ explained large reductions of *Streptococcus sobrinus* as being caused by an interference of micellar casein with microbial adhesion of oral bacteria. Lactoferrin, another milk constituent, also reduces adherence of *S. mutans* to the pellicle.^11,22^ In the present study, we focused on antimicrobial, anti-biofilm activities, but did not include demineralisation of dental hard tissues. This question was evaluated in several other in-vitro studies, which failed to reveal any cariogenic effect of bovine milk.^1,15,20,23^ This finding can be explained by the high buffering power of bovine milk.^15^ In addition to its high buffering capacity, bovine milk contains whey protein, minerals, and proteose-peptone fractions 3 and 5, which can prevent demineralisation of hard dental tissues.^11,36-37^

The fat content of dairy products seemed to play an important role in reducing cariogenicity. In a caries model, only whole milk but not skim milk maintained a pH above the demineralisation threshold.^10^

Neither low-fat nor high-fat yogurt affected the cariogenic biofilm and inhibited growth of *S. mutans* (clinical isolate), *S. sobrinus* and *L. acidophilus* only in very high concentrations. This is in contrast to a recent study, which reported a strong antibacterial activity against *S. mutans* strains.^32^ As these bacteria are aciduric, a pH effect of the yogurt might be excluded, as well as an effect by the bacteria incorporated into the yogurt which did not themselves interfere with bacterial growth of the caries-associated bacteria.^39^ However, it must be borne in mind that in-vitro data on plain yogurt’s effect on formation of a cariogenic biofilm are not available. The lack of anti-biofilm activity in the present study may raise the question about a potential replacement of the cariogenic bacteria by the yogurt bacteria; however, our analysis counted only very few of them in the biofilms (i.e. not more than 1%).

The growth of bacteria associated with periodontal disease was not suppressed by milk, but it was by yogurt, with no clear difference between low-fat and high-fat yogurt. This might be attributed to the different pH of milk and yogurt. The pH of milk is between 6 and 8, and the pH of yogurt 4.3 to 4.5.^21,25^ The lower pH of yogurt might lead to an unfavourable environment for periodontopathogenic species, which prefer alkaline milieu.^8^ On the other hand, the growth of all included *P. gingivalis* strains, *T. forsythia,* and *F. nucleatum* was inhibited by *L. delbrueckii ssp. bulgaricus*. This confirms a recent study, in which *L. delbruckii* isolated from dairy products were able to inhibit the growth of *P. gingivalis*.^4^ This suggests production of bacteriocins by *L. delbrueckii ssp. bulgaricus*. Lactobacilli (incl. *L. delbrueckii*) have been found to inhibit growth of opportunistic bacteria such as *Pseudomonas aeruginosa, Escherichia coli* and *Staphylococcus aureus*.^16^ Bactericidal activity depends on the growth conditions, and a pH close to pH 7.0 neutralises this activity.^19^

Periodontal biofilm formation was somewhat retarded by yogurt. Specifically, low-fat yogurt produced a trend toward slower periodontal biofilm formation, but the high-fat yogurt resulted in statistically significantly lower cfu counts. This result was supported by the analysis of metabolic activity, which showed striking differences. Further, although the cfu counts of a pre-formed biofilm were not affected by the yogurt, the metabolic activity of the biofilms after adding yogurt was low. Since cfu counts were not affected by yogurt, a modification of the biofilm composition might not be plausible. An explanation might be a downregulation of the metabolic processes within the biofilm. Acquiring more detailed information on bacterial composition and bacterial mRNA expression of the biofilm should be a goal of further studies. A single-species biofilm of *P. gingivalis* was inhibited by a milk constituent, lactoferrin.^6^ Lactoferrin does not influence the growth of *P. gingivalis,* but it does inhibit the proteolytic activity of *P. gingivalis*.^6^

The high-fat milk, low-fat and high-fat yogurt decreased the metabolic activity of Candida biofilm. Short- and medium-chain fatty acids have been shown to play important roles in antimicrobial activity. About one-third of the fatty acids in bovine milk are short- and medium-chain fatty acids. The major antimicrobial mechanisms were found to be cell-membrane destruction by physicochemical processes in Gram-positive bacteria and inhibition of microbial signal transduction and transcription.^7^ This might explain the lower metabolic activity of the Candida biofilms incubated with high-fat milk vs to low-fat milk in the present study. This effect might be higher for Candida biofilm compared to the other biofilms, because this biofilm is composed of only three species, while the other biofilms consisted of 5 and 6 different species.

In addition to an antimicrobial effect, milk and its fermented products exert anti-inflammatory activity on cells of the oral cavity. The expression of IL1- and TNFα-induced proinflammatory cytokines was reduced in gingival fibroblasts when exposed to pasteurised human milk, pasteurised bovine milk, as well as yogurt, buttermilk, sour milk, whey and powdered milk. Pasteurised milk and whey products were able to achieve a similar response in epithelial cells.^27^ Pasteurised bovine milk, yogurt, sour milk, and buttermilk have been shown to contain 1-2 ng transforming growth factor TGF-β that itself possesses anti-inflammatory properties.^28^ In gingival fibroblasts, pasteurised bovine milk and fermented milk products induce TGF-β target genes that appear to have protective functions in colitis.^24^ Furthermore, pasteurised human and bovine milk were able to induce the polarisation of macrophages from a pro-inflammatory M1 towards a pro-resolving M2 phenotype.^26^

Despite its strengths, this study also has some limitations. Milk and yogurt were made under standardised conditions. The multi-species biofilm models used defined strains. However, although several strains were used, the complexity of an oral biofilm is not reflected. Further, the focus was exclusively on oral microorganisms and biofilms and did not consider a potential immune response.

A possible therapeutic approach would be to recommend including yogurt, especially high-fat yogurt, to the diet of patients suffering from periodontitis. This is supported by the results of the present study, in which the in-vitro incubation of the biofilm and preformed biofilm decreased the metabolic activity of periodontopathogenic microorganisms with low-fat and high-fat yogurt; furthermore, high-fat yogurt decreased periodontopahogenic microorganisms in-vitro. Including yogurt in the diet could also help prevent periodontitis. Whether used therapeutically or prophylactically, the yogurt should contain no added sugar. Yogurt containing added sugar may increase the patient’s caries risk.

## Conclusion

The present in-vitro data have shown a potential effect of yogurt on the prevention and therapy of periodontal diseases and Candida infections. Thus, further studies are warranted, with the aim to verify in more detail the molecular basis of these effects and evaluate the interactions between oral microorganisms and host-derived cells.
